# Lncrna CASC11 aggravates diabetic nephropathy via targeting FoxO1

**DOI:** 10.5937/jomb0-42345

**Published:** 2023-08-25

**Authors:** Yun Zhang, Shuhan Shi, Changda Lin, Lishuang Che, Yuangen Li, Quanzuan Zeng, Weiyuan Lin

**Affiliations:** 1 Second Affiliated Hospital of Fujian Medical University, Department of Renal Medicine, Quanzhou, China

**Keywords:** Diabetic nephropathy (DN), CASC11, FoxO1, TGF-b1/Smads, dijabetička nefropatija (DN), CASC11, FoxO1, TGF-b1/Smads

## Abstract

**Background:**

To explore the biological effects of CASC11 on aggravating diabetic nephropathy (DN) by regulating FoxO1 (forkhead transcription factor O1).

**Methods:**

Serum levels of CASC11 and FoxO1 in DN patients were detected. The possibility of CASC11 in predicting the onset of DN was analyzed by depicting ROC curves. Correlation between CASC11 and FoxO1 was evaluated by Pearson correlation test. After intervening CASC11 and FoxO1 levels, we found that changes in proliferative and migratory abilities in high glucose (HG)induced kidney mesangial cells were determined respectively. Protein levels of TGF-β1 and Smads regulated by both CASC11 and FoxO1 were examined by Western blot.

## Abstract (sr)

**Uvod:**

Cilj je bio da se istraži biološki efekat CASC11 na pogoršanje dijabetičke nefropatije (DN) regulacijom FoxO1 (faktor transkripcije viljuške O1).

**Metode:**

Detektovani su nivoi CASC11 i FoxO1 u serumu kod pacijenata sa DN. Mogućnost CASC11 u predviđanju početka DN analizirana je prikazivanjem ROC krivih. Korelacija između CASC11 i FoxO1 je procenjena Pirsonovim korelacionim testom. Nakon intervenisanja nivoa CASC11 i FoxO1, otkrili smo da su određene promene u proliferativnim i migratornim sposobnostima u mezangijalnim ćelijama bubrega izazvanim visokom glukozom (HG). Nivoi proteina TGF-b1 i Smads regulisani i CASC11 i FoxO1 su ispitani Vestern blotom.

**Rezultati:**

Visoka ekspresija CASC11, ali niska ekspresija FoxO1 bili su u serumu pacijenata sa DN, što pokazuje da su negativna korelacija. Dakle, CASC11 može biti dijagnostički marker za DN. On je oslabio proliferativne i migratorne sposobnosti mezangijalnih ćelija bubrega izazvanih HG, ali gornji inhibitorni efekti CASC11 mogu se ukinuti prekomernom ekspresijom FoxO1. Osim toga, nivoi proteina TGF-b1 i Smads-a su pozitivno regulisani od strane CASC11, ali je regulacija Smads-a preokrenula takve promene.

**Zaključak:**

Kroz aktiviranje TGF-b1/Smads signalizacije, CASC11 inhibira ekspresiju FoxO1 i na taj način indukuje pogoršanje DN.

## Introduction

Diabetic nephropathy (DN) is one of the most serious comorbidities in diabetes patients, which is also a main cause of diabetes-induced death [1]. The incidence and prevalence of DN have shown upward trends in recent years. It is estimated that the number of people living with diabetes will reach 693 million globally by 2045 [2]. Sustained hyperglycemia will cause extensive vascular damages to eyes, kidneys, heart, and nerves. Approximately 40% of diabetes patients have a tendency to develop DN [3]. Therefore, timely prevention and treatment of diabetes and DN are of great significance.

Long non-coding RNAs (lncRNAs) are transcripts exceeding 200 nt long. Their structures are similar to those of mRNAs. However, lncRNAs lack cis-regulation ability and open reading frame [4]. Abnormal expression of lncRNA CASC11 has a relation to human diseases. It is reported that IL-9- induced activation of CASC11 accelerates the onset of arteriosclerosis via mediating apoptosis and proliferation in vascular smooth muscle cells [5] [6] [7]. During tumor progression, CASC11 is capable of regulating malignant phenotypes of tumor cells, indicating that it may be a promising biomarker for tumors diagnosis and prognostic evaluation [8]. However, the role of CASC11 in DN was few reported.

FoxO1 (forkhead transcription factor O1) exerts regulatory effects on gene transcription, anti-oxidative stress and metabolism [9]. Overexpression of FoxO1 is able to alleviate damage of podocytes in DN [10]. By high glucose induction, TGF-β1/Smad signaling drives epithelia-mesenchymal transition in podocytes, manifesting as downregulated nephrin (epithelial cell indicator) and upregulated desmin (interstitial cell indicator). In addition, TGF-β1/Smad signaling results in integrity destruction of glomerular filtration barrier and the occurrence of proteinuria, thereafter triggering the occurrence of DN [11]. LncRNA ANCR promotes migration and invasiveness in gastric cancer by regulating FoxO1 expression and suppressing M1 polarization of macrophages [12]. In a previous study, CTBP1-AS2 alleviates oxidative stress and inflammation induced by high-glucose through miR-155-5p/FOXO1 axis in diabetic nephropathy [13]. However, the relationship between CASC11 and Foxo1 in DN were unclear. In our research, CASC11 was hypothesized to aggravate diabetic ne phro pathy via targeting FoxO1, we aim to uncover the potential influences of CASC11 and FoxO1 on DN progression by collecting clinical samples of DN and generating *in vitro* HG model in kidney mesangial cells. Our findings provide novel ideas in health management of DN.

## Materials and methods

### Baseline characteristics

DN (n=50) and T2DM (n=50) patients were respectively enrolled in the DN and control group, respectively. T2DM and DN were diagnosed based on the WHO-1999 and WHO-2007 criteria [14] [15]. The diagnosis was confirmed by two professors. Candidates with T1DM or other types of diabetes, abnormal liver function, renal disease history, pregnancy, infectious diseases, thyroid diseases and those unwilling to be recruited were excluded. Venous blood samples were collected from them in anticoagulant tubes. After storage at 4°C for 30 min, it was centrifuged at 3,000 rpm for 15 min. The upper layer serum was collected and preserved at -80°C. The study was approved by the Ethical Committee of Second Affiliated Hospital of Fujian Medical University. Each participant provided the signed written informed consent.

### Cell culture and induction

Kidney mesangial cells preserved in the liquid nitrogen were recovered at 37°C. After centrifugation at 1,500 rpm for 5 min, the cells were cultured in Ros - well Park Memorial Institute 1640 (RPMI 1640) (pH 7.2) (Beyotime, Shanghai, China) containing 10% fetal bovine serum (FBS) (Beyotime, Shanghai, China), 100 μg/mL streptomycin and 100 μg/mL penicillin. They were passaged until 80% confluence. Kidney mesangial cells were induced in NG (normal glucose, 4.0 mmol/L) or HG (high glucose, 40.0 mmol/L), respectively.

### Cell transfection

Cells were cultivated in antibiotic-free medium for cell transfection. 0.5 μg plasmid (si-CASC11: 5’- GCCCACATCAAGCCTTCAT-3’; si-FoxO1: 5’-CCAGAUGCCUAUACAAACA- 3’) and 1 μL of Lipofectamine 2000 kit (Sigma-Aldrich; Merck KGaA) was respectively diluted in 50 μL of serum-free medium, which were mixed together and applied for cell transfection. Complete medium was replaced at 4–6 h, and transfected cells were cultivated another 36-h for further experiments.

### Quantitative real-time polymerase chain reaction (qRT-PCR)

Total RNA was extracted using TRIzol® reagent (Invitrogen; Carlsbad, CA, USA) according to manufacturer’s protocol. Total RNA was reverse transcribed into cDNA using the PrimeScript RT reagent kit (Invitrogen; Carlsbad, CA, USA), according to the manufacturer’s protocol. Levels were calculated using the method of 2-^ΔΔCt^. Primers were synthesized by XinFan Bio (Nanjing, China). Sequences of primers used for qRT-PCR were as follows: CASC11: 5’- CGACCCCAACACCTTCTTTG-3’ (forward) and 5’- CTCACCCCTAAGTYCGCTGG-3’ (reverse); FoxO1: 5’-ATGGTCAAGAGCGTGCCC-3’ (forward) and 5’- GATTGAGCATCCACCAAG-3’ (reverse); GAPDH: 5’- ACTGCCACCCAGAAGACT-3’ (forward) and 5’- GCTCAGTGTAGCCCAGGAT-3’ (reverse).

### Cell counting kit-8 (CCK-8)

The 96-well plates were inoculated at 2× 10^3^/well with 6 replicates in each group. 10 μL CCK-8 (Keygen, Nanjing, China) solution was added to each well and incubated for another 2-h. After 1-h incubation in the dark, absorbance at 450 nm was recorded at the indicated time points using the CCK 8 kit.

### Transwell assay

50 μL Fibronectin (FN) was added to the lower chamber and 100 μL matrix was added to the upper chamber. Cells (1×10^6^) were added to the upper chamber. The Transwell chamber was incubated in an incubator for 24h and then removed and placed in a 24-well plate, 500 μL methanol was added and fixed overnight at 4°C. After being washed with phosphate buffered saline (PBS) for three times, images were captured using an inverted microscope (Type: AZ100, Nikon, Tokyo, Japan).

### Western blot

Total proteins in each group of cells were extracted using RIPA (radioimmunoprecipitation) protein lysate (Beyotime, Shanghai, China). The extracted proteins were separated using a 12% sodium dodecyl sulphate-polyvinylidene polyacrylamide gel electrophoresis (SDS-PAGE) and transferred on to polyvinylidene fluoride membranes (Millipore, USA). Subsequently, the membranes were immersed in 5% skim milk for 2 hours. Primary antibodies were incubated for overnight incubation at 4°C. The next day, the membranes were incubated with horse radish peroxidase (HRP)-labeled secondary antibody for 2 h. Bands were exposed using electrochemiluminescence (ECL) reagent. Glyceraldehyde 3-phosphate dehydrogenase (GAPDH) was served as the internal control.

### Subcellular distribution analysis

1×10^6^ cells were lysed using Lysis Buffer. After being centrifugated using a centrifuge, the supernatant and precipitate were separated. Buffer SK and absolute ethanol were then added to them, and cytoplasmic RNA and nuclear RNA were extracted by column centrifugation.

### Statistical analysis

Statistical analysis were performed with the use of SPSS (Version X; IBM, Armonk, NY, USA). Differences between two groups were analyzed by using the Student’s *t*-test if they were normally distributed. Diagnostic value of CASC11 in DN was evaluated by depicting receiver operating characteristic (ROC) curves. All the experiments were repeated triple times. *P*<0.05 was considered as statistical significance.

## Results

### Comparison of baseline characteristics

By analyzing baseline characteristics between groups, we found no significant differences in age, gender, BMI, SBP, DBP, FBG, TC, HDL-C, LDL-C, TG and HbA1c were identified, indicating the two groups have a certain comparable value (*P*>0.05). In particular, values of Scr and BUN were observably higher in DN group compared with those of control group (*P*<0.05, Table 1).

**Table 1:** Comparison of baseline characteristics.

Variable	Control group	DN group	t	p
Age	53.18±7.75	54.75±7.92	1.002	0.319
Sex (male/female)	25/25	25/25	–	–
BMI (kg/m^2^)	26.35±3.32	26.73±3.01	0.600	0.550
SBP (mmHg)	131.31±19.27	131.93±19.82	0.159	0.874
DBP (mmHg)	83.64±9.07	84.32±9.78	0.360	0.719
FPG (mmol/L)	8.25±2.42	8.82±2.88	1.071	0.287
TC (mmol/L)	4.14±1.12	4.09±1.03	0.232	0.817
HDL-C (mmol/L)	1.41±0.52	1.45±0.63	0.346	0.730
LDL-C (mmol/L)	2.37±1.28	2.46±1.79	0.289	0.773
TG (mmol/L)	1.62±0.53	1.78±0.83	1.149	0.253
Scr (μmol/L)	54.31±15.72	63.12±19.11	2.518	0.013
BUN (mmol/L)	5.25±1.45	7.33±1.54	6.953	<0.001
HbA1c (%)	5.17±0.83	5.31±0.11	1.182	0.240

### Increased serum level of CASC11 in DN patients

**Figure 1:**
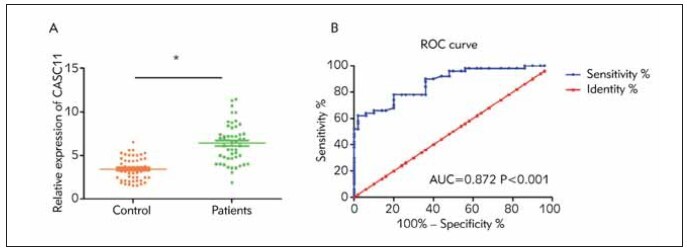
Increased serum level of CASC11 in DN patients. (A) Serum level of CASC11 increased in DN patients compared with that in controls; (B) ROC curves demonstrated the diagnostic potential of CASC11 in DN (AUC=0.872, P<0.001).

Compared with control group, serum level of CASC11 was observably higher in DN group (Figure 1A). Furthermore, ROC curves showed the diagnostic potential of CASC11 in DN (AUC = 0.872, 95%CI = 0.805–0.939, *P*<0.001, Figure 1B).

### CASC11 promotes the proliferative and migrative abilities in kidney mesangial cells

**Figure 2:**
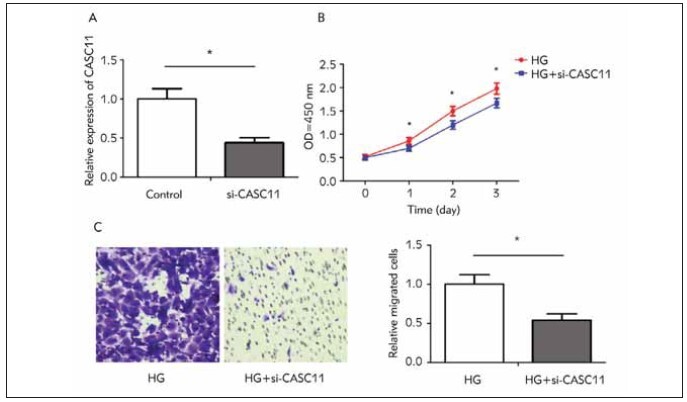
CASC11 promoted proliferative and migratory abilities in kidney mesangial cells. (A) Transfection of si-CASC11 significantly downregulated CASC11 in kidney mesangial cells; (B) CCK-8 assay showed inhibited proliferation in HG-induced kidney mesangial cells with CASC11 knockdown; (C) Transwell assay showed inhibited migration in HG-induced kidney mesangial cells with CASC11 knockdown. (magnification: 40×)

To uncover the role of CASC11 in the development of DN, we tested the transfection efficacy of si- CASC11 at first (Figure 2A). Knockdown of CASC11 markedly weakened viability and migratory ability in HG-induced kidney mesangial cells (Figure 2B, Figure 2C). It is suggested that CASC11 was able to enhance proliferative and migratory abilities in kidney mesangial cells induced by high glucose.

### CASC11 inhibited FoxO1 level

**Figure 3:**
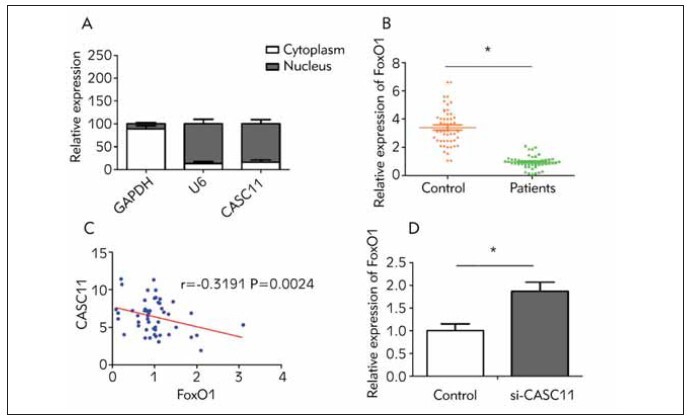
CASC11 inhibited FoxO1 level. (A) CASC11 mainly distributed in the nucleus; (B) Serum level of FoxO1 decreased in DN patients compared with that in controls; (C) CASC11 was negatively correlated to FoxO1 (r=-0.3191, P=0.0024); (D) FoxO1 was upregulated in HG-induced kidney mesangial cells with CASC11 knockdown.

Examination of the subcellular distribution revealed that that CASC11 was predominantly expressed in the nucleus (Figure 3A). Subsequently, serum level of FoxO1 in DN patients and T2DM patients was detected. Serum level of FoxO1 was much lower in DN patients compared with that of T2DM patients, and it was negatively correlated to CASC11 level (Figure 3B, Figure 3C). It is indicated that FoxO1 may be involved in the progression of DN as well. Furthermore, knockdown of CASC11 upregulated FoxO1, which supports a negative interaction between them (Figure 3D).

### CASC11 regulated kidney mesangial cell functions by FoxO1

**Figure 4:**
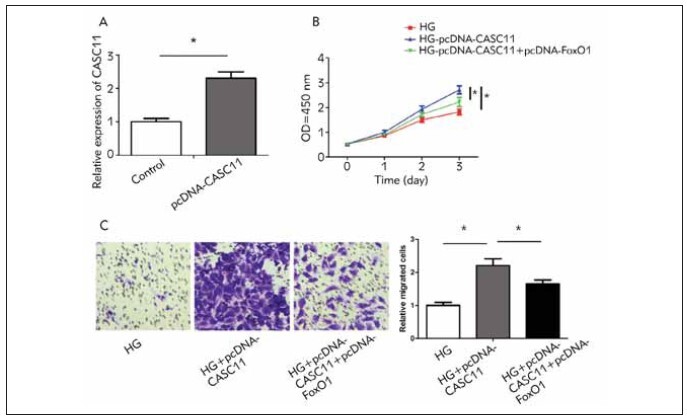
CASC11 regulated kidney mesangial cell functions by FoxO1. (A) Transfection of pcDNA-CASC11 significantly upregulated CASC11 in kidney mesangial cells; (B) Enhanced proliferation in HG-induced kidney mesangial cells overexpressing CASC11 was reversed by co-overexpression of FoxO1; (C) Enhanced migration in HG-induced kidney mesangial cells overexpressing CASC11 was reversed by co-overexpression of FoxO1 (magnification: 40×).

Transfection of pcDNA-CASC11 effectively upregulated CASC11 in kidney mesangial cells (Figure 4A). Overexpression of CASC11 enhanced viability and migratory cell number in HG-induced kidney mesangial cells, and the increased trends were reversed by co-overexpression of FoxO1 (Figure 4B, Figure 4C). Hence, FoxO1 was involved in CASC11-regulated functions of kidney mesangial cells.

### CASC11 negatively regulated FoxO1 level via the TGF-β1/Smads signaling

**Figure 5:**
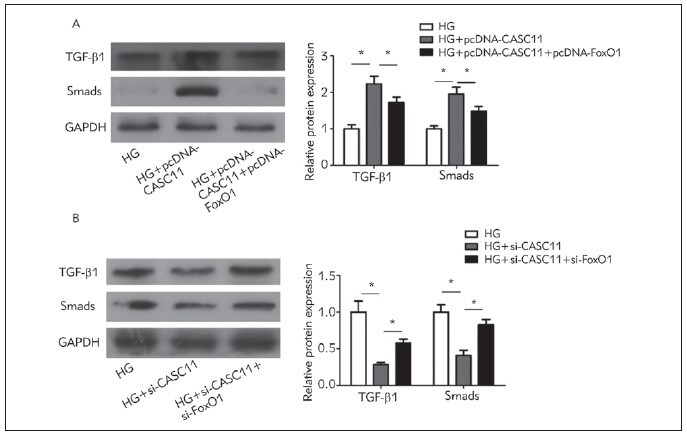
CASC11 negatively regulated FoxO1 level via the TGF-β1/Smads signaling. (A) Overexpression of FoxO1 reversed the promotive effects of CASC11 on protein levels of TGF-β1 and Smads in kidney mesangial cells; (B) Knockdown of FoxO1 reversed the inhibitory effects of CASC11 on protein levels of TGF-β1 and Smads in kidney mesangial cells.

As western blot analyses uncovered, protein levels of TGF-β1 and Smads were observably upregu lated in HG-induced kidney mesangial cells over expressing CASC11 compared with those of HG-in duced controls. Interestingly, the upregulated levels of TGF-β1 and Smads were downregulated by co-overexpression of FoxO1 (Figure 5A). As expected, downregulated TGF-β1 and Smads in HG-induced kidney mesangial cells with CASC11 knockdown were elevated by cosilence of FoxO1 (Figure 5B). Therefore, the TGF-β1/ Smads signaling was responsible for the regulatory effect of CASC11 on FoxO1 level.

## Discussion

Increased glomerular filtration capacity and microalbumin in the urine are the typical features of DN. If not treated in time, aggravated lesions, including glomerular atrophy, basement membrane thickening and extracellular matrix accumulation, eventually lead to chronic renal insufficiency [16]. Timely intervention for DN will decrease the outflow of microalbumin and thus prevent the development of proteinuria phase. Once DN patients are deteriorated in the proteinuria phase, their kidney functions will be severely impaired. Therefore, it is particularly important to develop therapeutic strategies of DN in the early phase [17].

LncRNAs are distributed in both cytoplasm and nucleus. Nuclear lncRNAs act on chromatin and regulate gene expressions, whereas cytoplasmic ones are able to regulate gene translation by acting on mRNAs [18]. Potential influences of lncRNAs on the development of DN have been identified. It is reported that lncRNA MALAT1 is upregulated in the *in vivo* T2DM model. Knockdown of MALAT1 remarkably alleviates T2DM-induced microvascular dysfunction (i.e. diabetic retinopathy) [19]. Gupta et al. [20] showed that expression levels of inflammatory factors (IL-6, IL-1β and TNF-α) increase at transcriptional and translational levels along with the upregulation of MALAT1 in HG-induced mouse renal endothelial cells. LncRNA CASC11 is located on chromosome 8q24.21 with 872 bp in transcripts, which is also known as cancer susceptibility candidate 11 or LINC00990 [21]. Zhang et al. [22] suggested that CASC11 is upregulated in colorectal cancer specimens. By targeting hnRNP-K, CASC11 drives malignant phenotypes in colorectal cancer cells via activating the WNT / β-catenin signaling. Our study demonstrated that CASC11 was highly expressed in the serum of DN patients, suggesting a certain diagnostic potential in DN. Moreover, CASC11 could stimulate proliferative and migratory abilities in kidney mesangial cells, and upregulate protein levels of TGF-β1 and Smads.

FoxO1 is a vital regulator in glycolipid metabolism, oxidative stress and cell functions [23]. Recent studies have uncovered that some Fox members, such as FoxM1, FoxC2 and FoxF2, are capable of intervening EMT [24] [25]. The differentiation of FoxO1 knockout mouse embryos is terminated in embryonic stage, indicating a relation between FoxO1 and cell differentiation in the pathological state [25]. The interaction between Smad protein and FoxO1 via the TGF-β signaling initiates the growth suppressor gene p21Cip1 [26]. In our experiment, FoxO1 was lowly expressed in the serum of DN patients, and negatively correlated to CASC11 level. Over-expression of FoxO1 reversed the modulatory functions of CASC11 on proliferative and migratory abilities in kidney mesangial cells, and the increase in protein levels of TGF-β1 and Smads. As a result, FoxO1 was responsible for CASC11 in regulating functions of kidney mesangial cells, and the TGF-β1 / Smads signaling.

Numerous tissue-specific lncRNAs have been found with the extensive application of sequencing and lncRNA microarray analyses. They are promising biomarkers for prevention, diagnosis and treatment of human diseases. Our findings suggested that lncRNA CASC11 could be utilized as a novel biomarker for DN.

## Conclusions

Through activating the TGF-β1/Smads signaling, CASC11 inhibits FoxO1 expression and thus induces aggravation of DN.

## Dodatak

### Funding

Natural Science Foundation of Fujian Province (2021J01250).

### Conflict of interest statement

All the authors declare that they have no conflict of interest in this work.
